# Managing co-morbid depression and anxiety in primary care patients with asthma and/or chronic obstructive pulmonary disease: study protocol for a randomized controlled trial

**DOI:** 10.1186/1745-6215-13-6

**Published:** 2012-01-11

**Authors:** Antoinette M Pommer, François Pouwer, Johan Denollet, Victor JM Pop

**Affiliations:** 1 Department of Medical Psychology & Neuropsychology, Center of Research on Psychology in Somatic diseases (CoRPS), Tilburg University, (Warandelaan), Tilburg, (P.O. Box 90153), The Netherlands

**Keywords:** Asthma, Chronic Obstructive Pulmonary Disease, Anxiety, Depression, Collaborative Care, Disease Management, Primary Care

## Abstract

**Background:**

Chronic Obstructive Pulmonary Disease (COPD) and asthma are common chronic diseases that are frequently accompanied by depression and/or anxiety. However, symptoms of depression and anxiety are often not recognized and therefore not treated. Currently, only a few studies have tested new clinical approaches that could improve the treatment of co-morbid depression and anxiety in these groups of patients.

**Methods/design:**

The present randomized controlled study will be conducted within the framework of PoZoB (Praktijk Ondersteuning Zuid-Oost Brabant), a large primary care organization in the Netherlands. Patients with asthma/COPD and co-morbid anxiety/depression will be included in order to test the effectiveness of a disease management approach to treat these co-morbid disorders. Important elements of this approach are: 1) systematic screening to improve detection of anxiety and depression 2) treatment in case of positive screening 3) monitoring of anxiety and depression 4) intensified treatment in case of non-remission (stepped care).

**Discussion:**

The present study is a large primary care study on the treatment of co-morbid depression and anxiety in patients with asthma and COPD. Strengths of this study are its randomized design, the focus on implementation in primary care and the fact that it applies the latest findings on the treatment of depression and anxiety. First results are expected in 2012/2013.

**Trial registration:**

Netherlands Trial Register (NTR): NTR2626

## Background

Chronic Obstructive Pulmonary Disease (COPD) and asthma are common chronic diseases, with a worldwide prevalence of around 9 to 10% [1] for COPD and 1 to 18% for asthma [2]. Although they represent two distinct entities, the disease processes and physical symptoms of both diseases overlap and can provoke a considerable burden on the daily lives of affected patients. The impact of these chronic conditions however, is not only characterized by a substantial physical burden, but also by the frequent occurrence of co-morbid affective problems like depression or anxiety [3].

### Depression and Anxiety in Asthma and COPD

The prevalence of depression and anxiety in patients with COPD varies widely between studies [4-6]. Recently Zhang et al. did a systematic review, meta-analysis and meta-regression on the prevalence of depressive symptoms in patients with COPD; only studies including a healthy control group were eligible. They found that patients with COPD have a higher prevalence of depressive symptoms than healthy controls (24,6%, 95% CI: 20.0 - 28.6% vs. 11,7%, 95% CI: 9-15.1%) [7]. For anxiety such a meta-analysis has not been conducted yet, however there is a review by Yohannes et al. on depression and anxiety in chronic heart failure and COPD. In their review Yohannes et al. report rates between 6 and 74% for self-reported symptoms of anxiety [5]. In asthma there is less variation in the prevalence rates for depression (around 10%) and anxiety (between 12 to 50) [8,9].

Asthma and COPD patients with co-morbid depression and/or anxiety tend to report an overall worse health status [5,10]. In addition, prospective research in patients with COPD showed that higher depression scores were associated with higher exacerbation frequencies (for symptom-based exacerbations: adjusted IRR, 1.51; 95% CI, 1.01-2.24; for event-based exacerbations: adjusted IRR, 1.56; 95% CI, 1.02-2.40) [10], a higher risk of hospitalization during follow-up (adjusted IRR, 1.72; 95% CI, 1.04-2.85) [10] and higher mortality rates (hazard ratio, 1.93; 95% CI, 1.04-3.58 and OR, 2.74; 95% CI, 1.42-5.29, respectively) [11,12] while anxiety was associated with increased overall length of exacerbations (1.92 times longer CI, 1.04-3.54) [10]. Moreover, anxiety and depression may even be more decisive predictors of functional capacity in patients with COPD than physiological markers such as lung function (OR 1.13; 95% CI, 1.02-1.26; versus no significant result for FEV1) [13].

In asthma, depression appeared to be associated with less adequate medication use and a lower level of physical exercise [8,9,14], and anxiety with more asthma symptoms, higher medication use and a more frequent use of healthcare services [15]. The latter, in turn, might explain the negative association that has been reported between anxiety and inflammation of the airways. Anxiety, as suggested, might lead to more prescription and/or greater consumption of corticosteroids which might result in less inflammation of the airways [9,15-17].

Hence, co-morbid depression and anxiety in patients with asthma or COPD not only affect quality of life but also treatment outcomes. In clinical practice however, there is a considerable under-detection of anxiety and depression; less than half of the depressed and/or anxious patients are recognized [18]. In patients with respiratory illnesses, recognizing depression or anxiety is further complicated due to overlap in symptoms, which makes a differential diagnosis difficult [19] and results in inadequate treatment of co-morbid depression and anxiety.

Only a few studies have examined how these co-morbid disorders should preferentially be treated [6,20-23]. Most studies were conducted within secondary care settings and investigated the short-term effectiveness of one type of behavioral therapy (e.g. education, cognitive behavioral therapy (CBT), problem solving therapy (PST), or relaxation therapy) without monitoring of long term results [6,20,21]. A large primary care study on late life depression in a general population, however, showed that a collaborative care approach in which mood status is monitored, with subsequent intensification of treatment when necessary was more effective in treating depression than merely offering one type of treatment [24].

### The present study

These findings resulted in the establishment of the 'DiMaCoDeA-AC' study; a study on a Disease Management approach for Co-morbid Depression and Anxiety in primary care patients with asthma or COPD. Employing a randomized controlled trial design, the effectiveness of a newly developed disease management approach is tested. In order to investigate the effectiveness of this new approach for the treatment of depression and anxiety in patients with asthma or COPD, it is compared to usual asthma/COPD care (care as usual). The disease management approach contains four core elements: 1) systematic screening to improve detection of anxiety and depression 2) treatment in case of positive screening 3) monitoring of anxiety and depression and 4) intensified treatment in case of non-remission (stepped care). The study will last for two years, in which treatment is offered during the first year, the second year will function as follow-up to study long-term effects. To the best of our knowledge such an approach has not been studied yet in these patient populations in a primary care setting.

## Methods/Design

### Hypotheses

Based on the current literature, we hypothesize that a disease management approach will result in reduced symptoms of depression and anxiety, reduced disease specific distress, and improved lifestyle behaviors (e.g. smoking cessation, increased physical activity, better medication adherence) and quality of life when compared to care as usual.

### Setting

The study will be conducted in collaboration with PoZoB (Praktijk ondersteuning Zuid-oost Brabant), a large managed care organization in the south of the Netherlands, established in 2001. PoZoB supports approximately 200 general practitioners (GPs) in regulating the care of patients with a chronic disease in a structured, optimal manner. In order to achieve this, several disease specific management programs have been developed. The first program 'DiaZoB' started in 2005 and regulates the care of patients with type 2 diabetes mellitus [25,26]. Following the success of DiaZoB, the 'AsCoZoB' management program was initiated in 2008 to regulate the care for patients with asthma and/or COPD and, during that same year, steps were taken to develop a 'care program' that focuses on the mental health domain. Recently, PoZoB developed a new management program for patients with cardiovascular risks and heart failure, the 'CVRM/HF' management program. These programs give the GP's the opportunity to refer patients with diabetes, asthma, COPD, cardiovascular risks, heart failure and/or mild mental health problems to special primary care nurses within their own general practice.

### Subjects

For the present study, only patients included in the AsCoZoB management program are eligible for participation, further exclusion criteria are: age below 18, currently receiving treatment for depression and/or anxiety, being diagnosed with a psychiatric disorder, suicidal ideation and not being able to read or speak Dutch sufficiently. All eligible patients will be approached via mail and screened for depression and anxiety using valid and reliable instruments. Patients with elevated scores on these scales will be invited for a baseline interview to further clarify possible exclusion criteria and to determine whether they suffer from a mood/anxiety disorder or sub threshold depression/anxiety using the depression and anxiety sections of the 'Mini International Neuropsychiatric Interview' (M.I.N.I.) [27]. Following this interview, patients meeting inclusion criteria are further informed about the design of the study and asked to give written informed consent if willing to participate. Informed consent will be sought for a) using anonymous data from the questionnaires for reports and scientific publications and b) for informing the general practitioner about study results. After written informed consent, patients will be included in the study and randomized to either the disease management or the care as usual condition.

### Randomization

Randomization will be performed by an external agency using a computerized random number generator. After inclusion the interviewer will call the agency to open an envelope which contains the condition to which the patient is allocated. To obtain equal numbers in both conditions a block randomization design is chosen.

### Design

#### Intervention: Disease management for co-morbid anxiety and depression

Within a week after the baseline interview, patients allocated to the disease management condition will be invited to start with a stepped care program consisting of three consecutive steps and monitoring of results, since anxiety and depression often are recurrent conditions. The stepped care intervention starts with four sessions of extensive psycho-education in which general information on anxiety and/or depression (e.g. their prevalence and signals) and their relation to asthma or COPD will be provided, supplemented with the rationale of cognitive behavioral therapy and practical tools to cope with symptoms of anxiety and/or depression. In case of non-remission of symptoms, patients will enter step two, consisting of a course on coping with depression and/or anxiety, depending on a patients need. Earlier research has demonstrated that this type of treatment is effective in reducing psychological distress in patients with asthma or COPD [20,28-31]. The course on anxiety was developed by the institute of mental health and addiction (Trimbos) in the Netherlands and has proven to be successful in reducing anxiety [32]. The course will be offered to individual patients and consists of ten consultations of approximately 30 minutes. Main elements are cognitive restructuring through the principals of cognitive behavioral therapy, behavioral activation, social skills training, and relapse prevention by discussing how to recognize and react on the first symptoms of depression/anxiety [33]. In case of non-remission of symptoms after step two, patients will enter step three: coaching complemented with anti-depressant and/or anxiolytic medication. Coaching entails six booster sessions from the courses described in step two. Medication is optional and will be prescribed by the general practitioner in accordance with the guidelines of the Dutch College of General Practitioners (Nederlands Huisartsen Genootschap standaard).

**Figure 1:**
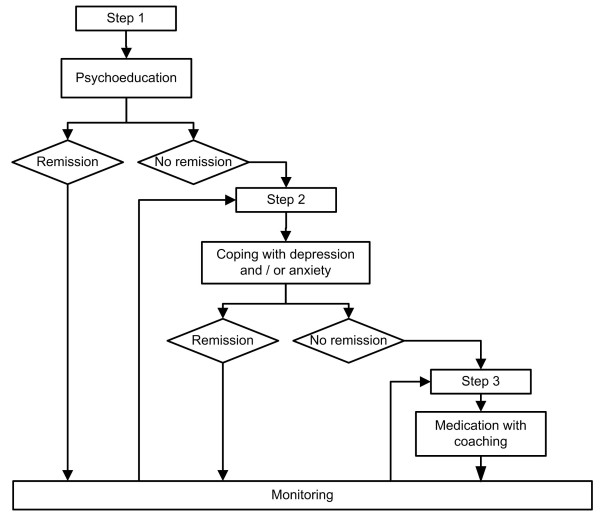
**Schematic outline of the stepped care program**.

Apart from the prescription of medication in step three, all these steps will be performed by trained primary care nurses. A schematic outline of the stepped care program is presented in Figure 1.

After each step (non-) remission is determined by monitoring of results using validated questionnaires. When further treatment is not indicated the emotional well-being of patients is monitored every three months for one year (using the PHQ-9 and the GAD-7). Patients are offered a next step in case of recurrent symptoms of anxiety or depression.

#### Care as usual

Within the care as usual condition no extra interventions are offered. Patients receive care as defined in the AsCoZoB management program (two annual visits with the primary care nurse to monitor disease progression), supplemented with a set of questionnaires including the PHQ-9 and GAD-7, every three months to record their psychological symptoms. Only if two consecutive scores on these questionnaires are equal to or above 15, or when scores indicate possible suicidal ideation, the GP is informed. However, patients can always consult their GP when necessary and receive treatment for depression/anxiety by the GP, or (after a referral) by a mental health specialist.

### Measurements

Both patients in the disease management and the care as usual condition will complete a set of questionnaires on seven occasions: T_0 _(baseline), T_1 _(3 months), T_2 _(6 months), T_3 _(9 months), T_4 _(12 months), T_5 _(18 months), T_6 _(24 months), of which T_0_, T_4 _and T_6 _will be done through interviewing. The instruments will consist of generic and asthma/COPD specific self-report questionnaires; table 1 presents an overview of variables measured at each time point. Moreover, at the baseline assessment, questions regarding demographic variables (e.g. age, marital status, work, educational level, socioeconomic status), psychiatric history (e.g. previous diagnosis of depression and/or anxiety, family history regarding psychiatric diagnosis) and/or health behaviors, (e.g. alcohol use, smoking habits, physical activity) will also be included. Clinical variables (FEV_1_/FVC and diagnosis of other chronic diseases will be obtained from medical records.

**Table 1:** variables measured at each time point

	FEV1	PHQ-9	GAD-7	**M.I.N.I**.	CCQ	ACQ	SF12
Time							
T_0_	X	X	X	X	X	X	X
T_1_		X	X				
T_2_		X	X				
T_3_		X	X				
T_4_	X	X	X	X	X	X	X
T_5_		X	X				
T_6_	X	X	X	X	X	X	X

#### Primary outcome measures

##### Depression and Anxiety

###### 1. The Patient Health Questionnaire-9 (PHQ-9)

The PHQ-9 is a short self-report questionnaire that is based on the nine symptoms of major depression as defined in the Diagnostic and Statistical Manual (DSM-IV). The PHQ-9 measures the presence of depressive symptoms in the past two weeks with four response options ('Not at all', 'Several days', 'More than half the days' and 'Nearly every day') and is often used in primary care settings. The scale has good overall accuracy, sensitivity and specificity in a general primary care population [34]. Lamers et al. recently did a study to determine what cut-off point of the PHQ-9 gave best sensitivity and specificity in patients with COPD, they found a cut-off of seven can best be used to indicate possible depression [35].

###### 2. The Generalized Anxiety Disorder-7 scale (GAD-7)

The GAD-7 was originally developed to screen for generalized anxiety disorder in primary care patients, however the scale has also shown good reliability and validity to detect other anxiety disorders [36]. The scale consists of seven items that, just as the items of the PHQ-9, are based on the DSM-IV criteria, responses range from 'not at all' to 'nearly every day'. A score above seven will be used to indicate a possible anxiety disorder.

###### 3. Mini International Neuropsychiatric Interview (M.I.N.I.)

The M.I.N.I. is a short diagnostic interview, also based on the DSM-IV criteria, that focuses on the existence of current psychiatric disorders [37]. The interview consists of separate modules to diagnose specific disorders. In this study, only the modules 'depression' and 'anxiety' will be administered to be able to diagnose past and current episodes.

##### Quality of life/Health Status

###### 1. Clinical COPD Questionnaire (CCQ)

The CCQ is a valid and reliable disease specific self administered questionnaire that measures "clinical control", which is 'the full range of clinical impairment that patients with COPD may experience as a result of their disease' [38]; the questionnaire is often referred to as a health status questionnaire. It was developed as a short questionnaire for use in everyday clinical practice and consists of 10 items, that can be answered on a seven point scale ranging from 'never' to 'almost all the time' and 'not limited at all' to 'totally limited/unable to do' [38].

###### 2. Asthma Control Questionnaire (ACQ)

The ACQ is a seven item, self administered, questionnaire developed to evaluate treatment effects by measuring asthma control, with good reliability and validity. All questions have a unique response scale with scores ranging from 'zero' to 'six', a high score always indicates poor control [39].

###### 3. Health Survey (SF-12)

The SF-12 is a health status questionnaire composed of 12 items which are derived from the 'Health Survey 36'. It has two components (mental and physical) that measure functional status, wellbeing and general health, higher scores indicate a better health status [40]. The scale was developed to use in a variety of chronic diseases including asthma and COPD [41,42].

### Ethical principles

The study will be conducted in accordance with the principles described in the Helsinki Declaration and in accordance with 'The Medical Research Involving Human Subjects Act' (WMO) and is approved by the medical ethics committee of the Elizabeth Hospital Tilburg, the Netherlands NL33363.008.10.

### Planned statistical analyses

Because it appears to be difficult to distinguish asthma and COPD in clinical practice, the decision was made to conduct the statistical analyses on the whole group of participants. All the data will be analyzed with the latest version of the 'Statistical Package for Social Sciences' (SPSS) and according to the intention-to-treat principle. First prevalence of depression and anxiety will be displayed in frequency tables and both conditions (control and intervention) will be compared using the Chi-square (Fisher's exact tests when appropriate) and Student's t-test, for discrete and continues variables respectively. Thereafter the effect of the intervention will be determined using multilevel analyses (mixed effect regression models) to compare baseline and follow up measures of all continuous data (scores on the PHQ-9, GAD-7, SF-12, CCQ and ACQ). In all these analyses a '*p*-value' of less than 0.05 will be considered statistically significant.

### Sample size calculation

In this study the change in scores on the PHQ-9 and GAD-7 are chosen as primary outcome measures to determine the effect of a disease management approach. Therefore, when calculating the required sample size, the desired difference in scores between the intervention and control condition on these scales was considered as starting point. A difference of 0.5 standard deviations was regarded necessary to find a clinically significant effect of the intervention. In order to detect this difference and assuming a power of 80% a minimum of 64 patients is needed in each condition [43]. When assuming that 20% of the participants drop-out, a number considered normal in this type of research, a minimum of 80 patients per condition is needed to maintain sufficient power. We expected that in order to reach this number, while anticipating a response-rate of 70% of which 20% is eligible, a total of 1142 patients will need to be screened.

## Discussion

This article describes the background, objectives and design of a large randomized controlled trial that will test the effectiveness of a stepped care program to treat co-morbid depression and anxiety in patients with asthma and/or COPD. Previous research in asthma and COPD has already shown that depression and anxiety are common co-morbidities in these patient populations and are negatively associated with functional capacity, health status, and health behaviors such as physical activity and positively with use of healthcare services, and health care costs [5,6,8-12,14-17]. However, randomized studies on the effects of treating depression and anxiety are scarce, mostly conducted in secondary care and often focusing on the effects of one type of therapy. Therefore, this study was developed to test the effectiveness of a disease management approach for co-morbid depression and anxiety in these patient populations. Important elements of this approach are: 1) systematic screening to improve detection of anxiety and depression using validated screening tools since symptoms of depression and anxiety are often not recognized and therefore remain untreated [18] 2) treatment in case of positive screening 3) monitoring of anxiety and depression and 4) intensified treatment in case of non-remission (stepped care). Other strengths of DiMaCoDeA-AC include the large sample size and its implementation in clinical practice to determine its effectiveness. However, the design of the study also has some limitations. Because the study will be conducted in practices of GP's who are already involved in a mental health program, there is a chance that the usual care is already relatively well organized with respect to co-morbid depression and/or anxiety. Another limitation is the choice to put asthma and COPD together in the statistical analyses since differentiating asthma and COPD often proves difficult in clinical practice. That does not mean however, that there are no differences between patients with asthma and COPD, patients with asthma, for example, will, on average, be younger than patients with COPD.

## Conclusions

In conclusion, asthma and COPD are both common chronic diseases that may provoke a considerable burden of disease, the current literature indicates that this burden of disease is often further increased by co-morbid anxiety and affective problems like depression. However, research into possible treatment strategies to alleviate this additional burden is limited. Previous reports on treatment of depression and anxiety suggest that a collaborative care approach may be the most beneficial. Therefore, the DiMaCoDeA-AC study tests the use of this approach to treat co-morbid depression and anxiety in patients with asthma and COPD. First results are expected in 2012/2013 and are expected to shed more light on the effect of monitoring and treating depression and anxiety in these patient populations.

## Trial status

The DiMaCoDeA-AC trial was conceived and designed in 2010. At the time this manuscript was submitted full approval by the Medical Ethics Committee had been obtained. The first participants were randomized in January 2011.

## List of abbreviations

COPD: Chronic Obstructive Pulmonary Disease; DiMaCoDeA-AC: Disease Management of Co-morbid Depression and Anxiety in patients with Asthma or COPD; PoZoB: Praktijk Ondersteuning Zuid-Oost Brabant; DiaZoB: Diabetes Zuid-Oost Brabant; AsCoZoB: Asthma COPD Zuid-Oost Brabant; CVRM/HF: Cardio Vascular Risk Management/Heart Failure; GP: General Practitioner; FEV1: Forced Expiration Volume at the end of the first second; FVC: Forced Vital Capacity; CBT: Cognitive Behavioral Therapy; PST: Problem Solving Therapy; DSM-IV: Diagnostic and Statistical Manual fourth edition; M.I.N.I.: Mini International Neuropsychiatric Interview; PHQ-9: Patient Health Questionnaire 9; GAD-7: Generalized Anxiety Disorder 7; CCQ: Clinical COPD Questionnaire; ACQ: Asthma Control Questionnaire; SPSS: Statistical Package for Social Sciences; IRR: Incidence Rate Ratios; CI: Confidence Interval; OR: Odds Ratio.

## Competing interests

The authors declare that they have no competing interests.

## Authors' contributions

FP and VP in collaboration with JD and AP designed the study. All authors have been involved in writing this manuscript and have approved the final manuscript and its submission.

## Funding

This study is funded by ZonMw, the Netherlands Organisation for Health Research and Development, DMCZ project 300020015 and is co-finded by C*o*RPS, Tilburg University.
